# NDM-1 encoded by a pNDM-BJ01-like plasmid p3SP-NDM in clinical *Enterobacter aerogenes*

**DOI:** 10.3389/fmicb.2015.00294

**Published:** 2015-04-14

**Authors:** Zhenhong Chen, Hongxia Li, Jiao Feng, Yuxue Li, Xin Chen, Xuemin Guo, Weijun Chen, Li Wang, Lei Lin, Huiying Yang, Wenhui Yang, Jie Wang, Dongsheng Zhou, Changting Liu, Zhe Yin

**Affiliations:** ^1^Nanlou Respiratory Diseases Department, Chinese People's Liberation Army General HospitalBeijing, China; ^2^State Key Laboratory of Pathogen and Biosecurity, Beijing Institute of Microbiology and EpidemiologyBeijing, China; ^3^The First Hospital of Shijiazhuang CityShijiazhuang, China; ^4^Zhongshan School of Medicine, Sun Yat-Sen UniversityGuangzhou, China; ^5^Beijing Institute of Genomics, Chinese Academy of SciencesBeijing, China

**Keywords:** *Enterobacter aerogenes*, NDM-1, Plasmid, p3SP-NDM

## Abstract

A carbapenem-nonsusceptible *Enterobacter aerogenes* strain named 3-SP was isolated from a human case of pneumonia in a Chinese teaching hospital. NDM-1 carbapenemase is produced by a pNDM-BJ01-like conjugative plasmid designated p3SP-NDM to account for carbapenem resistance of 3-SP. p3SP-NDM was fully sequenced and compared with all publically available pNDM-BJ01-like plasmids. The genetic differences between p3SP-NDM and pNDM-BJ01 include only 18 single nucleotide polymorphisms, a 1 bp deletion and a 706 bp deletion. p3SP-NDM and pNDM-BJ01 harbor an identical Tn*125* element organized as IS*Aba125, bla*_NDM−1_, *ble*_MBL_, Δ*trpF, dsbC, cutA*, Δ*groES, groEL, ISCR27*, and IS*Aba125*. The *bla*_NDM−1_ surrounding regions in these pNDM-BJ01-like plasmids have a conserved linear organization IS*Aba14-*aphA6*-Tn*125*-unknown* IS, with considerable genetic differences identified within or immediately downstream of Tn*125*. All reported pNDM-BJ01-like plasmids are exclusively found in *Acinetobacter*, whereas this is the first report of identification of a pNDM-BJ01-like plasmid in *Enterobacteriaceae*.

*Enterobacter aerogenes* is a Gram-negative bacterium widely found in the human gastrointestinal tract and in the environment, and generally non-pathogenic to healthy humans. Since 1990s, *E. aerogenes* has become an important opportunistic pathogen commonly affecting those with weakened immune systems to cause hospital-acquired infections such as pneumonia, bacteremia, urinary tract infection, surgical site infection, and meningitis (Georghiou et al., 1995; Davin-Regli et al., 1996; De Gheldre et al., 1997; Jalaluddin et al., 1998; Ronveaux et al., 1999).

*E. aerogenes* strains isolated from hospitalized patients generally exhibit high resistance to commonly used broad-spectrum antibiotics; in particular, the use of carbapenems imipenem and meropenem as the first-line antimicrobial agents for treating serious or refractory infections has led to considerable increase in prevalence of carbapenem-resistant *E. aerogenes* (De Gheldre et al., 1997; Chen et al., 2008; Lavigne et al., 2013). Carbapenem resistance of *E. aerogenes* is usually a result of production of plasmid-encoding carbapenemases such as KPC (Chen et al., 2014; Kuai et al., 2014; Luo et al., 2014; Qin et al., 2014), IMP (Biendo et al., 2008; Ding et al., 2014), VIM (Biendo et al., 2008; Souli et al., 2008) and NDM (Ho et al., 2012), decreased membrane permeability (due to altered porin expression or efflux pump overexpression) together with production of AmpC-type cephalosporinase or extended-spectrum β-lactamase (ESBL) (Bornet et al., 2003; Lavigne et al., 2012, 2013), and lipopolysaccharide modification (Leying et al., 1991).

The 47.27 Kb plasmid pNDM-BJ01 is isolated from a clinical *A. lwoffii* strain in China in 2010 (Hu et al., 2012) and it cannot be assigned into any known incompatibility group. In this study, phenotypic and biochemical experiments combined with plasmid sequencing and comparative genomics analyses disclose that production of NDM-1 by a pNDM-BJ01-like conjugative plasmid p3SP-NDM accounts for carbapenem resistance of a clinical *E. aerogenes* isolate recovered from a human case of pneumonia in a Chinese teaching hospital.

## Materials and methods

### Bacterial strains and identification

The use of human specimens and all related experimental protocols were approved by the Committee on Human Research of indicated institutions and carried out in accordance with the approved guidelines, and moreover the informed consent was obtained from indicated patient. All the bacterial strains in this study were subjective for species identification by BioMérieux VITEK 2, Bruker MALDI Biotyper, and 16s rRNA gene sequencing. For determination of 16S rRNA gene sequence, the almost complete coding region of 16S rRNA gene was amplified by PCR with the universal primers 27f (AGAGTTTGATCCTGGCTCAG) and 1492r (TACCTTGTTACGACTT) (Frank et al., 2008). The major carbapenemase and ESBL genes as listed in Table S1 were subjected to PCR detection. All PCR amplicons were sequenced on ABI 3730 Sequencer with the same primers for PCR.

### Plasmid transfer

Plasmid conjugal transfer experiments were carried out with the rifampin-resistant *E. coli* EC600 being used as recipient and the *bla*_NDM_-positive strain 3-SP as donor. 3 ml of overnight culture of each of donor and recipient bacteria were mixed together, harvested, and resuspended in 80 μl of Brain Heart Infusion (BHI) broth (BD Biosciences). The mixture was spotted on a 1 cm^2^ filter membrane that was placed on BHI agar (BD Biosciences) plate, and then incubated for mating at 37°C for 12–18 h. Bacteria were washed from filter membrane and spotted on Muller-Hinton (MH) agar (BD Biosciences) plate containing 750 μg/ml rifampin and 200 μg/ml ampicillin for selection of *bla*_NDM_-positive *E. coli* transconjugants.

To prepare competent cells for plasmid electroporation, 200 ml of overnight culture of *E. coli* DH10B in Super Optimal Broth (SOB) at an optical density (OD_600_) of 0.4–0.6 was washed three times with electroporation buffer (0.5 M mannitol and 10% glycerol) and concentrated into a final volume of 2 ml. 1 μg of plasmid DNA, which was isolated from 3-SP with QIAGEN Plasmid Midi Kit, were mixed with 100 μl of competent cells for electroporation at 25 μF, 200 Ω, and 2.5 Kv. The resulting cells were suspended in 500 μl of SOB, and an appropriate aliquot was spotted on SOB agar plate containing 200 μg/ml ampicillin for selection of *bla*_NDM_-positive *E. coli* electroporants.

### S1-PFGE and southern blot

Bacterial genomic DNA was prepared in agarose plugs and digested with S1 nuclease (Takara). The linearized plasmids and partially digested genomic DNA were separated through the CHEF-Mapper XA PFGE system (Bio-Rad). The DNA fragments were stained with ethidium bromide (EtBr), transferred to a Hybond N^+^ membrane (GE Amersham Biosciences) and hybridized with a DIG-labeled probe specific to *bla*_NDM_ (Rasheed et al., 2013). Probe labeling and signal detection were carried out with DIG high primer DNA labeling and detection starter kit II according to the manufacturer's instructions (Roche Diagnostics).

### Detection of carbapenemase activity

Activity of class A/B/D carbapenemases was determined by CarbaNP test (Dortet et al., 2012) with modifications. Overnight bacterial cell culture in MH broth was diluted 1:100 into 3 ml of fresh MH broth, and bacteria were allowed to grow at 37°C with shaking at 200 rpm to reach an OD_600_ of 1.0–1.4. If required, ampicillin was used at 200 μg/ml. Bacterial cells were harvested from 2 ml of the above culture, and washed twice with 20 mM Tris-HCl (pH 7.8). Cell pellets were resuspended in 500 μl of 20 mM Tris-HCl (pH 7.8), and lysed by soniation, followed by centrifugation at 10,000 ×g at 4 °C for 5 min. 50 μl of the supernatant (the enzymatic bacterial suspension) were mixed with 50 μl of substrate I to V, respectively, followed by incubation at 37°C for a maximum of 2 h. Substrate I: 0.054% phenol red plus 0.1 mM ZnSO_4_ (pH 7.8). Substrate II: 0.054% phenol red plus 0.1 mM ZnSO_4_ (pH 7.8), and 0.6 mg/μl imipenem. Substrate III: 0.054% phenol red plus 0.1 mM ZnSO_4_ (pH 7.8), 0.6 mg/μl mg imipenem, and 0.8 mg/μl tazobactam. Substrate IV: 0.054% phenol red plus 0.1 mM ZnSO_4_ (pH 7.8), 0.6 mg/μl mg imipenem, and 3 mM EDTA (pH 7.8). Substrate V: 0.054% phenol red plus 0.1 mM ZnSO_4_ (pH 7.8), 0.6 mg/μl mg imipenem, 0.8 mg/μl tazobactam, and 3 mM EDTA (pH 7.8).

### Determination of minimum inhibitory concentration (MIC)

The MIC values of indicated bacterial strains were tested by using VITEK 2 according to manufacturer's instructions, and antimicrobial susceptibility was judged by Clinical and Laboratory Standards Institute (CLSI) standard.

### Determination of plasmid DNA sequence

The chromosome DNA-free plasmid DNA was isolated from the cell cultures of indicated *E. coli* transconjugant using a Qiagen large construct kit, and then sequenced by using whole-genome shotgun strategy in combination with Illumina HiSeq 2500 sequencing technology. The contigs were assembled with Velvet, and the gaps were filled through combinatorial PCR and Sanger Sequencing on ABI 3730 Sequencer. The genes were predicted with GeneMarkS and further annotated by BLASTP against UniPort and NR databases.

### Nucleotide sequence accession numbers

The complete sequence of plasmid p3SP-NDM was submitted to GenBank under accession number KP900015.

## Results

### Carbapenem-nonsusceptible *E. aerogenes* 3-SP

In June 2012, an 86-year-old male with cough and fever visited a teaching hospital in Xi'an city of China. The patient had underlying sequelae of cerebral hemorrhage, and complained of recurrent pulmonary infection. The patient received oral administration with cefradine for a week, but his symptoms did not improve. The patient was subsequently hospitalized, and chest X-ray examination confirmed presence of bilateral pulmonary infection and he was accordingly diagnosed to have pneumonia. The sputum specimens were sampled on the same day of admission. On the next day, round bacterial colonies were observed after cultivation of sputum on MH agar, and the bacterial isolate designated 3-SP was identified as *E. aerogenes* by VITEK 2, Bruker MALDI Biotyper, and 16s rRNA gene sequencing. The antimicrobial susceptibility test using VITEK 2 indicated 3-SP was resistant to multiple β-lactam antibiotics including imipenem and meropenem but remained susceptible to fluoroquinolones. The patient accordingly received intravenous administration with levofloxacin, and his symptoms associated with pulmonary infection disappeared and he was discharged after 10 days of antimicrobial treatment.

### NDM-producing plasmid p3SP-NDM

PCR detection of the major ESBL and carbapenemase genes (Table S1) indicated presence of only *bla*_NDM_ in *E. aerogenes* 3-SP (Figure S1), which was confirmed by PCR amplicon sequencing. A *bla*_NDM_-positive *E. coli* EC600 transconjugant named 3-SP-NDM-EC600 and a *bla*_NDM_-positive *E. coli* DH10B electroporant designated 3-SP-NDM-DH10B were obtained. The S1-PFGE/southern hybridization assay detected a ~48 kb plasmid in each of 3-SP, 3-SP-NDM-EC600 and 3-SP-NDM-DH10B, which could hybridize with a *bla*_NDM_-specific probe (Figure S2). The modified CarbaNP test showed that 3-SP, 3-SP-NDM-EC600, and 3-SP-NDM-DH10B had Ambler class B carbapenemase activity (Figure S3). The antibiotic susceptibility test showed that 3-SP, 3-SP-NDM-EC600, and 3-SP-NDM-DH10B were highly resistant to all the penicillin, β-lactamase, monobactam, cephalosporin, and carbapenem drugs tested, but remained to be susceptible to fluoroquinolones, furanes, aminoglycosides, and sulfanilamide tested (Table 1). The above results indicated that 3-SP contained a conjugative NDM-encoding plasmid (designated p3SP-NDM), which accounted for carbapenem resistance of 3-SP and could be transferred into and mobilized in *E. coli* recipients.

**Table 1:** **MIC values and antimicrobial susceptibility**.

**Category**	**Antibiotics**	**MIC (μg/ml)/antimicrobial susceptibility**				
		**3-SP**	**3-SP-NDM-EC600**	**3-SP-NDM-DH10B**	**EC600**	**DH10B**
Penicillin	Ampicillin	>=32/R	>=32/R	>=32/R	16/I	<=2/S
	Ampicillin/sulbactam	>=32/R	>=32/R	>=32/R	8/S	<=2/S
	Piperacillin	>=128/R	>=128/R	>=128/R	<=4/S	<=4/S
	Piperacillin/tazobactam	>=128/R	64/R	64/R	<=4/S	<=4/S
Monobactam	Aztreonam	>=64/R	>=64/R	>=64/R	<=1/S	<=1/S
Cephalosporin	Cefazolin	>=64/R	>=64/R	>=64/R	<=4/S	<=4/S
	Cefuroxime sodium	>=64/R	>=64/R	>=64/R	16/I	4/S
	Cefuroxime axetil	>=64/R	>=64/R	>=64/R	16/I	4/S
	Cefotetan	>=64/R	>=64/R	32/R	<=4/S	<=4/S
	Ceftriaxone	>=64/R	>=64/R	>=64/R	<=1/S	<=1/S
	Ceftazidime	>=64/R	>=64/R	>=64/R	<=1/S	<=1/S
Carbapenem	Imipenem	8/R	>=16/R	>=16/R	<=1/S	<=1/S
	Meropenem	8/R	4/R	8/R	<=0.25/S	<=0.25/S
Fluoroquinolone	Ciprofloxacin	2/I	<=0.25/S	<=0.25/S	<=0.25/S	<=0.25/S
	Levofloxacin	2/S	0.5/S	<=0.25/S	1/S	<=0.25/S
Furane	Macrodantin	64/I	<=16/S	<=16/S	<=16/S	<=16/S
Aminoglycoside	Amikacin	<=2/S	<=2/S	<=2/S	<=2/S	<=2/S
	Gentamicin	<=1/S	<=1/S	<=1/S	<=1/S	<=1/S
	Tobramycin	<=1/S	<=1/S	<=1/S	<=1/S	<=1/S
Sulfanilamide	Trimethoprim/sulfamethoxazole	<=20/S	<=20/S	<=20/S	<=20/S	<=20/S

*S, sensitive; R, resistant; I, Intermediate*.

**Figure 1:**
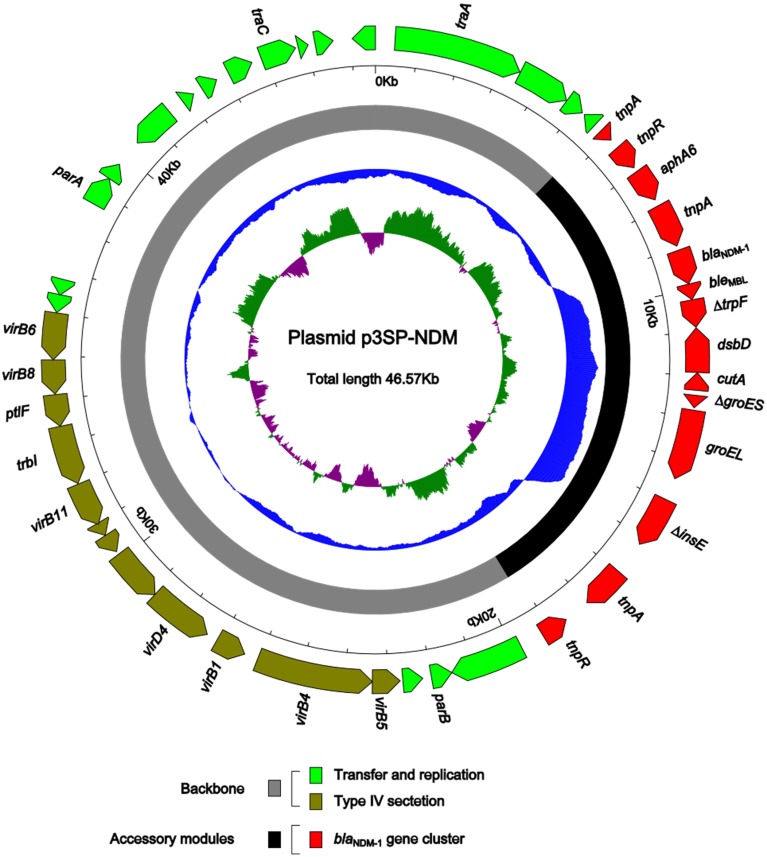
**Schematic map of p3SP-NDM**. Genes are denoted by arrows and colored based on gene function classification. The innermost circle presents GC-Skew [(G − C)/(G + C)] with a window size of 500 bp and a step size of 20 bp. The blue circle presents GC content. Shown also are backbone and accessory module regions. All the gene organization figures in this work were drawn by using the Inkscape software (https://inkscape.org/).

Plasmid DNA was isolated from 3-SP-NDM-EC600, and the whole genome sequence of p3SP-NDM was determined to 46,570 bp in length with a 137 fold coverage, forming a circular DNA sequence with a total of 45 open reading frames annotated (Figure 1).

### Comparative genomics of pNDM-BJ01-like plasmids

p3SP-NDM is highly similar to pNDM-BJ01 with genetic differences including only 18 single nucleotide polymorphisms and an 1 bp deletion (Table S2) and a 706 bp deletion (Figure S3, see also below).

**Figure 2:**
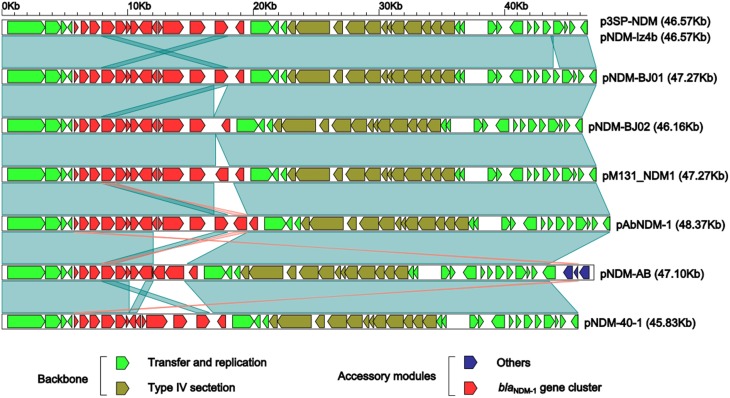
**Linear comparison of sequenced plasmids**. Genes are denoted by arrows and colored based on gene function classification. Dark green shading regions denote regions of homology (>99% nucleotide similarity).

Linear structural comparison (Figure 2) was performed with whole genome sequences of p3SP-NDM, pNDM-BJ01 and all the six additional pNDM-BJ01-like plasmids pNDM-BJ02 (Hu et al., 2012), pNDM-40-1 (Jones et al., 2014), pNDM-AB (Zhang et al., 2013), pNDM-Iz4b (KJ547696), pAbNDM-1 (JN377410), and pM131_NDM1 (JX072963) (collected from GenBank on November 20, 2014). p3SP-NDM and pNDM-Iz4b essentially had the same genomic organization.

The above eight plasmids contain a highly conserved backbone composed of two separate regions of plasmid replication/transfer and one region of type VI section system, with only one structural polymorphism that a 706 bp fragment (nucleotide position 43,861–44,566 in pNDM-BJ01; located within the plasmid replication/transfer region and contains only one annotated gene encoding hypothetical protein) is deleted from p3SP-NDM and pNDM-Iz4b relative to all the other plasmids (Figure 2).

As for accessory modules (Figure 2), each of these eight plasmids contains a *bla*_NDM−1_ gene cluster located around nucleotide position 5685; in addition, pNDM-AB harbors an additional 3.5 Kb accessory region, which is located around nucleotide position 5570 and composed of an IS*Aba14* element and a gene encoding type I restriction-modification system methyl transferase subunit.

**Figure 3:**
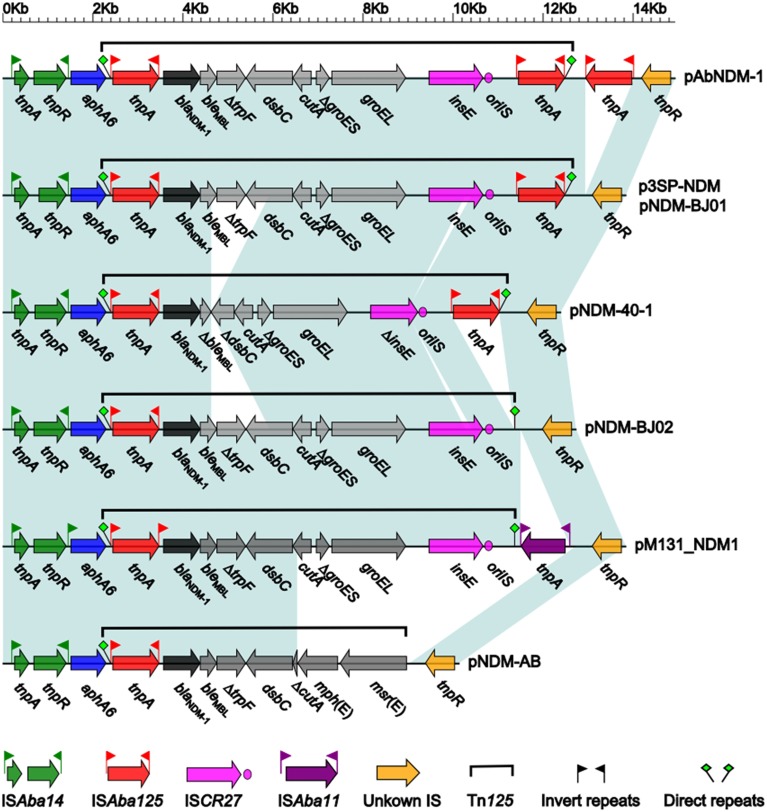
**Linear comparison of *bla*_NDM−1_ gene clusters**. Genes are denoted by arrows and colored. Dark green shading regions denote regions of homology (>99% nucleotide similarity).

The *bla*_NDM−1_ gene clusters from the above eight plasmids show a conserved linear organization IS*Aba14*-*aphA6*-Tn*125*-unknown IS, and the IS*Aba14*-*aphA6* and unknown IS fragments are essentially identical structurally in these plasmids while structural differences occur within or immediately downstream of the composite transposon Tn*125* (Figure 3). pNDM-BJ01 and p3SP-NDM contain the prototype Tn*125*, which is sequentially organized as IS*Aba125, bla*_NDM−1_, *ble*_MBL_ (bleomycin resistance), Δ*trpF, dsbC, cutA*, Δ*groES, groEL, ISCR27*, and IS*Aba125* (Figure 3); Tn*125* is inserted into a site downstream of *aphA6* (aminoglycoside resistance), which is evidenced by presence of GTT direct repeats at both ends, and the two copies of IS*Aba125* likely target *bla*_NDM−1_ surrounding sequences to promote formation and transposition of Tn*125* (Poirel et al., 2012).

Compared to the counterpart in pNDM-BJ01/p3SP-NDM, Tn*125* in pNDM-40-1 (Figure 3) is a truncated version with two deletions: a 1298 bp deletion within original *ble*_MBL_-Δ*trpF*-*dsbC* to generate Δ*ble*_MBL_-Δ*dsbC*, and a 150 bp deletion within IS*CR27* (Jones et al., 2014). A third copy of IS*Aba125* or an intact IS*Aba11* element is inserted immediately downstream of the intact Tn*125* of pAbNDM-1 or pM131_NDM1, respectively, while the downstream copy of IS*Aba125* is deleted from Tn*125* of pNDM-BJ02 (Figure 3). As for Tn*125* in pNDM-AB, *cutA*-Δ*groES*-*groEL*-*ISCR27* observed in pNDM-BJ01 is replaced by Δ*cutA*-*mph*(E)- *msr*(E), leading to absence of downstream GTT direct repeat (Zhang et al., 2013); by contrast, GTT direct repeats are intact in all other seven plasmids (Figure 3). The *mph*(E) and *msr*(E) genes confer macrolide/triamilide resistance (Michael et al., 2012).

## Discussion

NDM, initially identified in *Klebsiella pneumoniae* in 2009, is a metallo-β-lactamase (MBL) capable of hydrolyzing almost all clinically used β-lactams (Tiwari and Moganty, 2013), and the *bla*_NDM_ genes have been found in a large collection of Gram-negative bacteria of clinical, environmental and animal origins, especially including *Acinetobacter, Enterobacteriaceae*, and *Pseudomonas* (Nordmann et al., 2011; Johnson and Woodford, 2013; Dortet et al., 2014). Fourteen NDM variants have been described, differing by several amino acid changes, and a few of them have been tested for their enzymatic kinetics, which denotes that amino acid substitution is a major source of MBL activity extension (Nordmann et al., 2012; Tada et al., 2013). Nevertheless, a systematic characterization of enzymatic kinetics of all the identified NDM variants is needed.

Intact IS*Aba125* has never been found in bacterial species other than *Acinetobacter*, and thus IS*Aba125* ought to originate from *Acinetobacter*. *bla*_NDM−1_ is most likely generated in an *Acinetobacter* background by a fusion event between *aphA6* and an ancestral metallo-β-lactamase gene (Poirel et al., 2012; Toleman et al., 2012; Zong and Zhang, 2013). Insertion of various derivates of *bla*_NDM−1_-carrying Tn*125* have been found within *Acinetobacter* chromosomes (Pfeifer et al., 2011; Bonnin et al., 2012; Partridge and Iredell, 2012; Poirel et al., 2012) and plasmids (Hu et al., 2012; Partridge and Iredell, 2012; Zhang et al., 2013; Zong and Zhang, 2013; Jones et al., 2014) at different locations, and moreover Tn*125* derivates also represent plasmid-borne *bla*_NDM−1_ contexts in *Enterobacteriaceae* (Sekizuka et al., 2011; Mcgann et al., 2012; Partridge and Iredell, 2012; Fiett et al., 2014; Mataseje et al., 2014). These indicate emergency of *bla*_NDM−1_in *Acinetobacter* and then dissemination among *Enterobacteriaceae*. In addition, the upstream copy of IS*Aba125*, either intact or interrupted by other mobile elements, of Tn*125* provides *bla*_NDM_ with a strong promoter to drive high-level production of NDM enzymes (Poirel et al., 2011; Toleman et al., 2012).

At the time of writing this paper, there are at least eight additional pNDM-BJ01-like plasmids have been deposited in GenBank. All the above plasmids are exclusively found in *Acinetobacter* species including *A. lwoffii, A. baumannii, A. ereziniae, A. pittii*, and an unidentified *Acinetobacter* species from China, India, and Pakistan. This is the first report of identification of a pNDM-BJ01-like plasmid in *Enterobacteriaceae*, indicating spread of pNDM-BJ01-like plasmids from *Acinetobacter* to *Enterobacteriaceae*.

There is only one preliminary report describing detection of *bla*_NDM_ in *E. aerogenes*, and this strain harbors a ~50 Kb *bla*_NDM−1_-encoding plasmid and is recovered from the stool sample of a 1-year-old infant with cough and intermittent fever in Hunan Province of China (Ho et al., 2012). This work presents extended evidence that NDM-1 is produced by a conjugative 46.57 Kb plasmid p3SP-NDM, and accounts for carbapenem resistance of clinical *E. aerogenes*; phenotypic and biochemical experiments combined with plasmid sequencing and comparative genomics analyses give a deeper understanding of antibiotic resistance mechanism of this NDM-1-producing *E. aerogenes* strain.

### Conflict of interest statement

The authors declare that the research was conducted in the absence of any commercial or financial relationships that could be construed as a potential conflict of interest.

## Supplementary material

Figure S1 **PCR detection of *bla* genes**. The major ESBL and carbapenemase genes (Table S1) were screened by PCR with strains 3-SP, 3-SP-NDM-EC600, 3-SP-NDM-DH10B, ATCC BAA-2146 (a NDM-1-producing reference strain of *K. pneumoniae*, Rasheed et al., 2013), EC600, and DH10B. Of all the *bla* genes detected, only *bla*_NDM_ was shown to be present in each of 3-SP, 3-SP-NDM-EC600, and 3-SP-NDM-DH10B.

Figure S2 **S1-PFGE/Southern blot**. The S1-digested genomic DNA samples was analyzed on an EtBr-stained PFGE gel **(A)**, and then subjected to Southern blot hybridization with a DIG-labeled probe specific to *bla*_NDM−1_**(B)**.

Figure S3 **Detection of carbapenemase activity**. In the presence of any carbapenemase, relevant carbapenems are hydrolyzed and transformed into its carboxylic form, thus leading to a pH decrease which is detected by a color change of phenol red solution (red to yellow–orange). Ambler class A carbapenemases are, at least partially, inhibited by tazobactam, whereas class B carbapenemases (metallo-ß-lactamases) are inhibited by divalent cation chelators such as EDTA. There is no available chemical inhibitor for class D carbapenemases. In this study, the *bla*_NDM_-psitive strains 3-SP, 3-SP-NDM-EC600, 3-SP-NDM-DH10B, and ATCC BAA-2146 had class B carbapenemase activity. As expected, *E. coli* EC600 and DH10B had no carbapenemase activity.
