# Supplementary figures and images for: NDM-1 encoded by a pNDM-BJ01-like plasmid p3SP-NDM in clinical Enterobacter aerogenes
Source: Front Microbiol. 2015 Apr 14;6:294. doi: 10.3389/fmicb.2015.00294 (PMC4396501; doi:10.3389/fmicb.2015.00294)

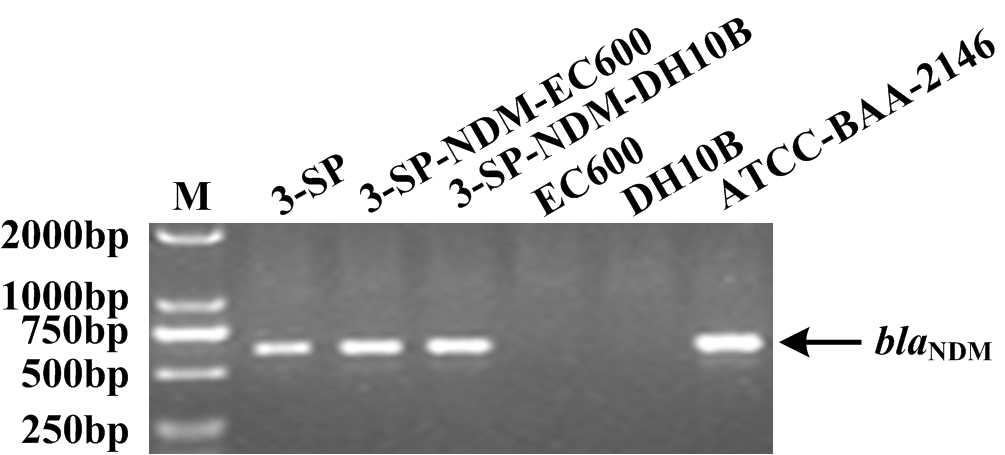

Supplement: Figure S1 — PCR detection of bla genes. The major ESBL and carbapenemase genes (Table S1) were screened by PCR with strains 3-SP, 3-SP-NDM-EC600, 3-SP-NDM-DH10B, ATCC BAA-2146 (a NDM-1-producing reference strain of K. pneumoniae, Rasheed et al., 2013), EC600, and DH10B. Of all the bla genes detected, only blaNDM was shown to be present in each of 3-SP, 3-SP-NDM-EC600, and 3-SP-NDM-DH10B. [file Image1.JPEG]

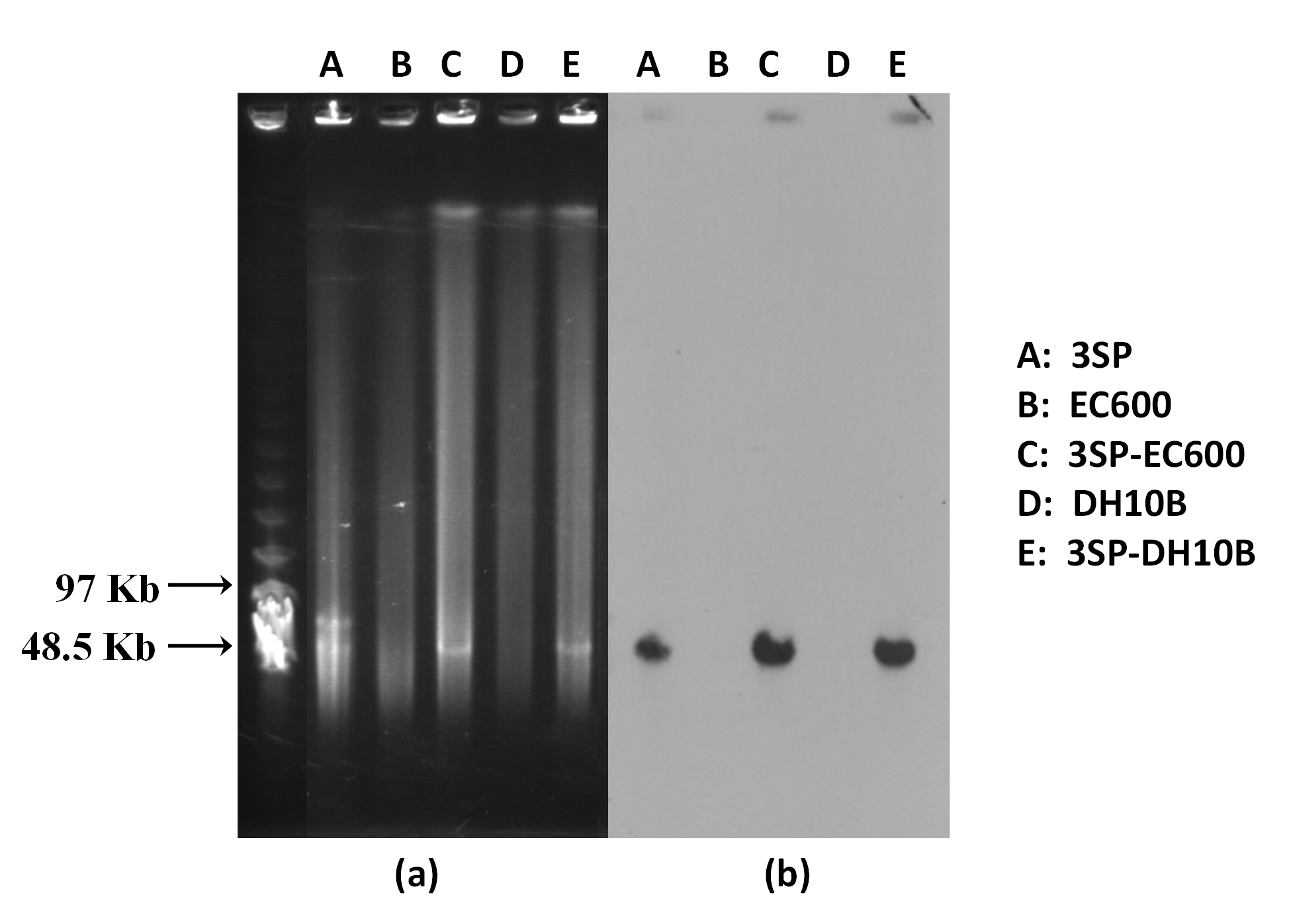

Supplement: Figure S2 — S1-PFGE/Southern blot. The S1-digested genomic DNA samples was analyzed on an EtBr-stained PFGE gel (A), and then subjected to Southern blot hybridization with a DIG-labeled probe specific to blaNDM−1(B). [file Image2.JPEG]

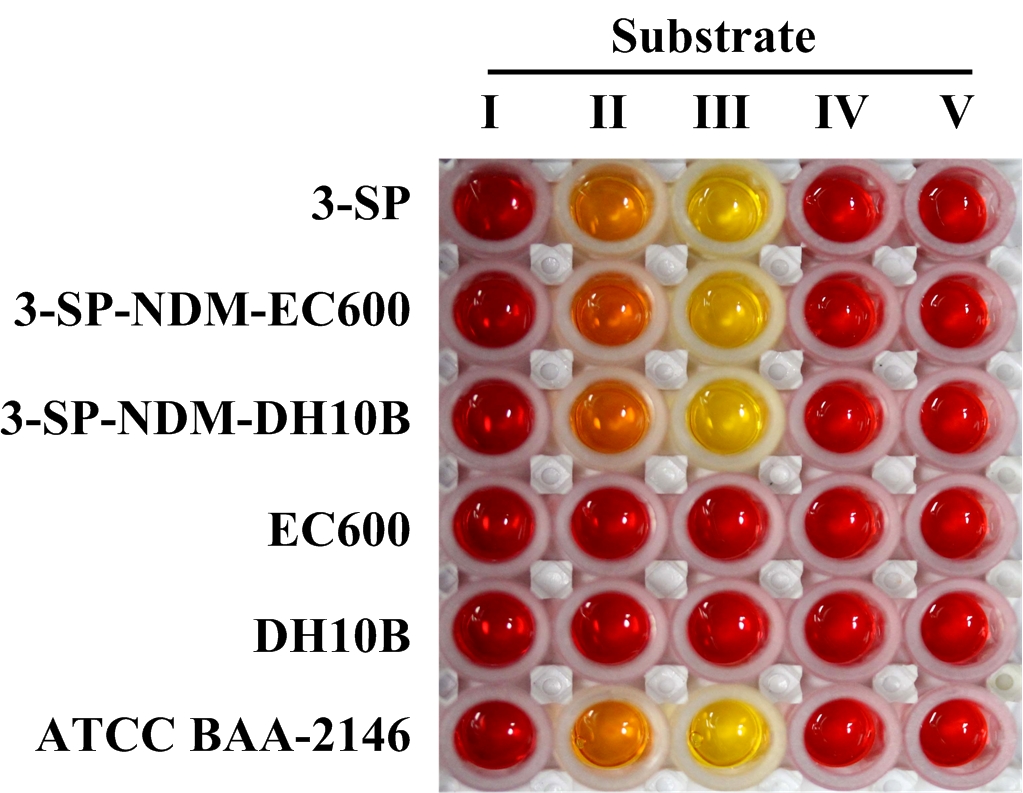

Supplement: Figure S3 — Detection of carbapenemase activity. In the presence of any carbapenemase, relevant carbapenems are hydrolyzed and transformed into its carboxylic form, thus leading to a pH decrease which is detected by a color change of phenol red solution (red to yellow–orange). Ambler class A carbapenemases are, at least partially, inhibited by tazobactam, whereas class B carbapenemases (metallo-ß-lactamases) are inhibited by divalent cation chelators such as EDTA. There is no available chemical inhibitor for class D carbapenemases. In this study, the blaNDM-psitive strains 3-SP, 3-SP-NDM-EC600, 3-SP-NDM-DH10B, and ATCC BAA-2146 had class B carbapenemase activity. As expected, E. coli EC600 and DH10B had no carbapenemase activity. [file Image3.JPEG]
