# Supplementary material for: NDM-1 encoded by a pNDM-BJ01-like plasmid p3SP-NDM in clinical Enterobacter aerogenes
Source: Front Microbiol. 2015 Apr 14;6:294. doi: 10.3389/fmicb.2015.00294 (PMC4396501; doi:10.3389/fmicb.2015.00294)
Supplement: Supplementary file 5 [file Table2.DOCX]

**Table S2 Genetic variations between p3SP-NDM and pNDM-BJ01**

| **p3SP-NDM** | | **pNDM-BJ01** | |
| --- | --- | --- | --- |
| **Position** | **Nucleotide** | **Position** | **Nucleotide** |
| 504 | A | G | 504 |
| 8328 | C | A | 8328 |
| 17688 | T | C | 17688 |
| 17760 | C | T | 17760 |
| 31694 | G | T | 31694 |
| 37189 | T | C | 41630 |
| 37258 | T | C | 41561 |
| 37273 | C | T | 41546 |
| 37282 | C | T | 41537 |
| 40343 | C | Absent | |
| 41538 | A | G | 37282 |
| 41547 | A | G | 37273 |
| 41562 | G | A | 37258 |
| 41631 | G | A | 37189 |
| 43782 | T | A | 44486 |
| 43816 | A | C | 43815 |
| 43840 | T | A | 43839 |
| 43846 | T | C | 43845 |
| 43848 | A | G | 43847 |
